# Assessing national nutrition security: The UK reliance on imports to meet population energy and nutrient recommendations

**DOI:** 10.1371/journal.pone.0192649

**Published:** 2018-02-28

**Authors:** Jennie I. Macdiarmid, Heather Clark, Stephen Whybrow, Henri de Ruiter, Geraldine McNeill

**Affiliations:** 1 The Rowett Institute, University of Aberdeen, Aberdeen, United Kingdom; 2 Institute of Applied Health Sciences, University of Aberdeen, Aberdeen, United Kingdom; 3 Institute of Biological and Environmental Sciences, University of Aberdeen, Aberdeen, United Kingdom; 4 Information and Computational Sciences Group, The James Hutton Institute, Aberdeen, United Kingdom; Wageningen University, NETHERLANDS

## Abstract

Nutrition security describes the adequacy of the food supply to meet not only energy but also macronutrient and micronutrient requirements for the population. The aim of this study was to develop a method to assess trends in national nutrition security and the contribution of imports to nutrition security, using the UK as a case study. Food supply data from FAO food balance sheets and national food composition tables were used to estimate the nutrient content of domestically produced food, imported food and exported food. Nutrition security was defined as the total nutrient supply (domestic production, minus exports, plus imports) to meet population-level nutrient requirements. The results showed that the UK was nutrition secure over the period 1961–2011 for energy, macronutrients and key micronutrients, with the exception of total carbohydrates and fibre, which may be due to the loss of fibre incurred by processing cereals into refined products. The supply of protein exceeded population requirements and could be met with domestic production alone. Even excluding all meat there was sufficient protein for population requirements. The supply of total fat, saturated fat and sugar considerably exceeded the current dietary recommendation. As regards nutrition security in 2010, the UK was reliant on imported foods to meet energy, fibre, total carbohydrate, iron, zinc and vitamin A requirements. This analysis demonstrates the importance of including nutrients other than energy to determine the adequacy of the food supply. The methodology also provides an alternative perspective on food security and self-sufficiency by assessing the dependency on imports to meet population level nutritional requirements.

## Introduction

Achieving global nutrition security is a major challenge driven by the need for healthy and sustainable diets to feed the growing global population and to address inequalities in the distribution of, and access to, food [1,2]. This challenge exists against a backdrop of climate change, diminishing natural resources and increasing affluence, which is changing dietary habits [3]. To date, the focus of food security has been almost exclusively on ensuring that there is a sufficient supply of energy for people, however, micronutrient deficiencies along with hunger and overnutrition are increasingly being recognised as a further requirement [4]. A wider approach is necessary to ensure that the supply of nutrients is sufficient to meet dietary requirements. One of the UN Sustainable Development Goals (SDG) is to end hunger and ensure food security and improved nutrition [5]. To achieve this there must be an adequate supply of nutrients at a national level to meet population requirements, which may come from domestic production or imported food.

Globalisation of the food system means that many countries are increasingly dependent on trade to supply sufficient food; it is estimated that 23% of food produced for human consumption is traded internationally [6]. The global food supply is sensitive to natural, social, political and economic disruptions and the consequences of climate change, all of which pose a threat to nutrition security [7]. A recent report also highlights the importance of infrastructure for global transportation of food, identifying major ‘chokepoints’ for supply of staple foods, including maritime corridors, coastal infrastructure and inland transportation systems [8]. Disruption at any of these points could have serious implications for transportation of food and hence nutrition security. The degree of self-sufficiency at a country level is often viewed in terms of risks of instability and disruption to global food supplies, and reducing these risks is therefore an important element of national food policy [9–11]. Reducing reliance on imported food and increasing domestic production has not only trade and economic implications but also potential nutritional consequences when a country is dependent on imports to meet the nutritional requirements of its population. Self-sufficiency is defined using a number of different indices (e.g. the economic value of commodities, volume of food or energy supply), which can give different perspectives of the situation, but self-sufficiency is rarely considered in term of nutritional adequacy [12]. In the UK, for example, self-sufficiency is estimated to be around 60% based on the monetary value of raw food commodities at the farm gate available for human consumption (i.e. ratio of food production to supply) [13], but this does not address whether the supply is nutritionally adequate for health. Porkka *et al*. [14] assessed global and national self-sufficiency in terms of energy supply and illustrated the increased importance of trade to meet national deficits of energy, but their analysis did not include other nutrients.

Domestic production, imports, exports and stock variations for the supply of a range of food commodities for human consumption are estimated at country level by the FAO in the food balance sheets (FBS). Energy, fat and protein available per capita are derived in the FBS, but these nutrients alone are not sufficient to determine nutrition security. Previous studies have added other nutrients to these calculations, but these have tended to be single nutrients, such as zinc [15–18]. More recently, Smith *et al*. [19] mapped a wider range of nutrients to the FBS at a global scale as part of the GENuS project. Beal *et al*. [20] also mapped nutrient data to the FBS data to estimate the global adequacy of micronutrient supplies. Nutrition security and self-sufficiency at a country level can be derived from FBS since they provide estimates of availability, utilisation and trends of the food supply from domestic production, imports and exports data.

The aim of this study was to develop a method to assess the adequacy of the supply of energy, macronutrients and micronutrients at a country level and to measure the UK dependency on imported food to meet population nutritional requirements since 1961. The UK was used as a case study since it represents a country with high levels of food imports and while energy intakes are sufficient, diets are associated with a high prevalence of diet-related chronic disease. As with many high-income countries, the consumption of meat is high and production of livestock is associated with high levels of greenhouse gas emissions, so dietary changes are needed to reduce the risk of climate change.

## Materials and methods

The FBS data (http://www.fao.org/economic/ess/fbs/en/) were used to assess the trend over time in UK nutrition security and dependency on imports to meet population energy, macronutrient and micronutrient requirements. The total supply of each food commodity in the FBS used for human consumption was derived from total domestic production (p), imports (i), exports (e) and variation in stock levels (ds). The supply of nutrients for human consumption were divided into three categories:

1. *Total food supply* available in the UK for human consumption = p + i − e + ds
2. Food supply from *domestic production* used for consumption in the UK = p − e + ds
3. Food *imported* into the UK for human consumption = i

### Nutrient composition of the food commodities

Data for energy, macronutrients and micronutrients were estimated by matching the foods in each of the FBS commodity group to foods in UK food composition tables, McCance & Widdowson version 7 (M&W) [21]. The nutrient dataset derived from the UK food composition tables comprised energy, protein, total fat, saturated fat, total carbohydrate, fibre (non-starch polysaccharides (NSP)), free sugars (total sugar minus sugar in whole fruit, vegetables, pulses and milk), vitamin A (retinol equivalents), vitamin B_12_, vitamin C, folate, calcium, zinc and iron. To investigate the balance of the whole food supply, current population-weighted nutrient recommendations with upper limits (i.e. fat, saturated fat and free sugars), as well as those with lower limits (i.e. protein, NSP, minerals and vitamins) were included. The micronutrients were selected either because they are in foods associated with recommended dietary changes (e.g. reduction in meat consumption) or because current intakes at population level or in subgroups of the population are marginal. The data included foods that are fortified with nutrients in the UK, in order to estimate the total nutrient supply at the point of consumption.

The method used to map nutrient data to food commodities followed the FAO approach to estimate energy, total fat and protein in the FBS [22]. FBS data report standardised food commodities available for human consumption (commodity level), which are an aggregate of individual food and food products (food level). For example, the nutrient data for the commodity level of ‘wheat and wheat products’ are based on the food level items of wheat flour and wheat derived products (e.g. macaroni, bread, breakfast cereals and pastry). The FBS provide a list of foods included in each commodity group [23] and these were matched with food items in the UK food composition tables. Food not typically eaten in the UK, such as camels and rodents, were not included. Seven hundred and seventy one foods at the food level were matched across 85 standardised FBS commodity groups. The FBS data are based on ‘as supplied’ weights rather than as eaten and only include waste prior to reaching the household (e.g. storage, transportation and retail, but not including household food waste). Therefore, the following adjustments were made to the data to convert the commodities to edible forms of foods:

1. *Raw commodities vs*. *foods as eaten*: Nutrients were calculated based on the edible portion of foods, rather than the raw commodity as reported in the FBS. Meat and fish were converted to the weight of only the edible portion (e.g. exclude bones), since they are reported as carcass weights [24]. In the case of fruit, vegetables and nuts the nutrient data were based on the edible portion e.g. without peel or shells.
2. *Weighting nutrient data for individual foods in the commodity group*: The FBS food commodities comprise a number of food items but the amount of each of the food item used in the estimation of the nutrient content of the commodity group is not reported. The foods in some of the commodity groups are typically eaten in different quantities and have very different nutrient compositions. For example, the commodity group ‘milk, excluding butter’ includes the food items milk, cheese, yoghurt and ice-cream, which not only have very different nutrient densities but tend to be purchased and consumed in different amounts. In these cases, a weighting was applied to reflect the amounts of each of these foods purchased in the UK, using household purchase data from the UK Living Costs and Food Survey [25].
3. *Household food waste*: The FBS data do not take into account household food waste. A waste factor for different foods in each commodity group was applied using UK household food waste data. Where data were unavailable for perishable foods, a value of 16% by weight was used, based on an average of all perishable foods [26].

Energy and all nutrients were estimated using UK food composition tables. To assess the accuracy of these estimated values, the outputs were compared with the energy, fat and protein reported in the FBS in 2010 (S1 Fig). The total supply of energy reported in the FBS was 3382kcal/capita/d compared with 3433kcal/capita/d estimated using the UK food composition data, with good agreement across the commodity groups. For total fat, the corresponding figures were 158g/capita/d and 165g/capita/d, while for protein the estimates were 101g/capita/d and 113g/capita/d in the FBS and UK, respectively.

### Trends in nutrient supply

The trends in nutrient supply, based on domestic production, imports, exports and variation in stocks, were estimated for each year from 1961 to 2011. The food sources of the nutrients in 1962 were compared with 2010, each based on an average of three years. For example, data labelled 1962 were the average of years 1961, 1962 and 1963 and data labelled 2010 were the average of 2009, 2010 and 2011. Data from the 85 food groups were aggregated into 14 wider food categories for presentation purposes (S1 Table).

### Population-weighted energy and nutrient requirements

Population-weighted energy and nutrient requirements were based on the current UK dietary reference values and recommendations, taking into account the different nutritional requirements of the population by age and sex. Requirements were weighted according to the distribution of age and sex of the population in each year to take account of changes over time in population demographics [27]. The estimated average requirement for energy at a population level was based on the amount of energy considered sufficient to meet the requirements of 50% of the population. This accounts for variation in physical activity by age and partly to allow for growth; the estimated energy requirements were calculated using 1.4xbasal metabolic rate (BMR) (age 1–3 years), 1.58xBMR (age 4–9 years), 1.75xBMR (age 10–18 years) and 1.63xBMR (age 19+) [28]. Reference nutrient intakes (RNI, the minimum amount considered adequate to meet nutrient requirements of 97.5% of healthy individuals) were used for protein, vitamin C, vitamin B_12_, folate, vitamin A (as retinol equivalents), calcium, iron and zinc [29]. Current recommended population average intakes for total fat, saturated fat, total carbohydrate and free sugars (total sugars minus sugar from whole fruit, pulses, vegetables and milk), expressed as a percentage of total energy supply, were used. Population-level intakes above the recommendation for total fat, saturated fat and free sugars are currently considered undesirable for health. Fibre (non-starch polysaccharides (NSP)) was expressed in grams per day [29,30].

## Results

### Food sources of energy and nutrients between 1962 and 2010

The comparison of food sources of energy and macronutrients from domestically produced food and imported food in 1962 (average of 1961, 1962 and 1963) and 2010 (average of 2009, 2010 and 2011) are shown in Table 1, and the food sources of micronutrients in Table 2. There was little change in the source of the energy over time, with the most noticeable difference being in the reduction of energy from animal fats and increase from vegetable oils. While the total supply of energy from cereals, root vegetables and sugar has remained relatively stable over time, a greater proportion of this energy is now coming from foods domestically produced than from imports. The reverse was true for commodities such as fruit, vegetables, milk and fish as imports of these have increased, which is reflected in the increasing contribution of imports to the supply of micronutrients (Table 2). Despite a shift from consumption of whole milk to lower fat milks over this period, the overall contribution of milk and milk products to total fat and saturated fat did not change between 1962 and 2010, suggesting that the fat removed from milk is being consumed in other food products. The total supply of fruit and vegetables changed over the decades. In 1962, the supply was equivalent to 335g/capita/d, of which 53% was imported, and this has steadily increased to 593g/capita/d in 2010, with 84% coming from imports, while the domestic supply slowly decreased from 176g/capita/day in 1962 to 94 g/capita/d in 2010.

**Table 1:** Comparison of the food sources supplying energy and macronutrients available for consumption in the UK from domestic production and imports (1962 and 2010).

		Energy (kcal/capita/d)		Protein (g/capita/d)		Total fat (g/capita/d)		Saturated fat (g/capita/d)		Total carbohydrates (g/capita/d)		Free sugars (g/capita/d)		Fibre (NSP) (g/capita/d)	
		Domestic	Import	Domestic	Import	Domestic	Import	Domestic	Imports	Domestic	Import	Domestic	Import	Domestic	Import
Cereals	1962	265.2	365.8	8.2	11.3	3.9	5.0	1.4	1.9	52.6	71.6	4.2	6.1	3.5	4.3
	*2010*	*498*.*8*	*187*.*0*	*15*.*7*	*4*.*9*	*7*.*5*	*1*.*8*	*2*.*7*	*0*.*6*	*98*.*5*	*34*.*1*	*8*.*2*	*1*.*7*	*6*.*7*	*1*.*5*
Starchy roots	1962	134.6	12.7	3.2	0.3	0.2	0.0	0.1	0.0	32.0	3.0	0.0	0.0	1.6	0.2
	*2010*	*116*.*0*	*36*.*4*	*2*.*8*	*0*.*9*	*0*.*2*	*0*.*0*	*0*.*1*	0.0	*27*.*6*	*8*.*7*	*0*.*0*	*0*.*0*	*1*.*4*	*0*.*4*
Sugars	1962	44.1	373.4	0.0	0.3	0.1	1.0	0.1	0.6	11.4	96.8	10.8	91.7	0.0	0.0
	*2010*	*105*.*8*	*222*.*2*	*0*.*1*	*0*.*2*	*0*.*3*	*0*.*6*	*0*.*2*	*0*.*3*	*27*.*5*	*57*.*6*	*25*.*9*	*54*.*5*	*0*.*0*	*0*.*0*
Pulses	1962	6.1	16.2	0.4	1.2	0.1	0.1	0.0	0.0	1.0	2.7	0.0	0.0	0.3	0.8
	*2010*	*12*.*8*	*6*.*2*	*0*.*9*	*0*.*5*	*0*.*1*	*0*.*0*	*0*.*0*	0.0	*2*.*2*	*1*.*0*	*0*.*0*	*0*.*0*	*0*.*5*	*0*.*3*
Nuts, seeds	1962	-1.9	35.4	0.0	0.7	-0.2	3.3	-0.1	2.0	0.0	0.7	0.0	0.4	0.0	0.6
	*2010*	*-4*.*6*	*62*.*6*	*0*.*0*	*1*.*8*	*-0*.*5*	*5*.*6*	*-0*.*2*	*1*.*9*	*-0*.*1*	*1*.*4*	*-0*.*1*	*0*.*6*	*-0*.*1*	*0*.*8*
Vegetable oil	1962	72.9	86.0	0.0	0.0	8.1	9.6	2.4	3.5	0.0	0.0	0.0	0.0	0.0	0.0
	*2010*	*176*.*4*	*191*.*8*	*0*.*0*	*0*.*0*	*19*.*6*	*21*.*3*	*1*.*5*	*3*.*8*	*0*.*0*	*0*.*0*	*0*.*0*	*0*.*0*	*0*.*0*	*0*.*0*
Vegetables	1962	19.1	8.5	1.2	0.4	0.3	0.1	0.1	0.0	3.0	1.7	0.0	0.0	1.5	0.4
	*2010*	*15*.*6*	*25*.*0*	*0*.*9*	*1*.*4*	*0*.*2*	*0*.*3*	0.0	*0*.*1*	*2*.*7*	*4*.*5*	*0*.*0*	*0*.*0*	*1*.*0*	*1*.*6*
Fruit (incl juice)	1962	9.8	49.4	0.1	0.7	0.1	0.2	0.0	0.0	2.3	11.9	0.6	2.0	0.3	1.0
	*2010*	*0*.*3*	*110*.*6*	*0*.*0*	*1*.*7*	*0*.*0*	*0*.*5*	*0*.*0*	*0*.*1*	*0*.*1*	*26*.*5*	*0*.*1*	*5*.*1*	*0*.*0*	*2*.*7*
Meat (incl offal)	1962	170.2	121.8	15.0	9.8	11.9	8.9	4.4	3.4	0.9	0.5	0.4	0.2	0.1	0.1
	*2010*	*147*.*5*	*146*.*1*	*15*.*0*	*13*.*4*	*9*.*5*	*10*.*0*	*3*.*4*	*3*.*6*	*0*.*5*	*0*.*6*	*0*.*2*	*0*.*3*	*0*.*1*	*0*.*1*
Animal fat	1962	94.8	261.8	0.1	0.1	10.4	28.8	4.9	14.8	0.3	0.5	0.1	0.1	0.0	0.0
	*2010*	*59*.*5*	*62*.*6*	*0*.*0*	*0*.*1*	*6*.*6*	*6*.*8*	*3*.*3*	*3*.*5*	*0*.*1*	*0*.*2*	*0*.*0*	*0*.*1*	*0*.*0*	*0*.*0*
Eggs	1962	39.0	2.3	3.7	0.2	2.7	0.2	0.7	0.0	0.0	0.0	0.0	0.0	0.0	0.0
	*2010*	*25*.*7*	*3*.*9*	*2*.*5*	*0*.*4*	*1*.*8*	*0*.*3*	*0*.*5*	*0*.*1*	*0*.*0*	*0*.*0*	*0*.*0*	*0*.*0*	*0*.*0*	*0*.*0*
Milk & products	1962	324.0	76.3	19.9	4.7	15.0	3.5	9.4	2.2	29.9	7.0	0.0	0.0	0.0	0.0
	*2010*	*278*.*8*	*134*.*1*	*17*.*1*	*8*.*2*	*12*.*9*	*6*.*2*	*8*.*1*	*3*.*9*	*25*.*7*	*12*.*4*	*0*.*0*	*0*.*0*	*0*.*0*	*0*.*0*
Fish	1962	15.3	9.0	3.1	1.3	0.3	0.4	0.1	0.1	0.0	0.0	0.0	0.0	0.0	0.0
	*2010*	*-0*.*7*	*25*.*7*	*-0*.*1*	*4*.*2*	*0*.*0*	*0*.*9*	*0*.*0*	*0*.*2*	*0*.*0*	*0*.*2*	*0*.*0*	*0*.*1*	*0*.*0*	*0*.*0*
Other	1962	65.2	45.1	0.54	0.7	-0.5	1.68	-0.3	1.0	2.9	5.0	4.1	4.7	0.0	0.1
	*2010*	*32*.*1*	*152*.*5*	*-0*.*09*	*2*.*2*	*-1*.*1*	*3*.*5*	*-0*.*6*	*2*.*0*	*-0*.*4*	*15*.*3*	*1*.*0*	*13*.*3*	*-0*.*1*	*0*.*3*
TOTAL	**1962**	***1258*.*2***	***1463*.*7***	***55*.*6***	***31*.*8***	***62*.*9***	***52*.*2***	***23*.*1***	***29*.*8***	***201*.*6***	***136*.*4***	***20*.*1***	***105*.*3***	***7*.*1***	***7*.*5***
	***2010***	***1464*.*1***	***1366*.*8***	***54*.*7***	***39*.*8***	***58*.*0***	***57*.*0***	***19*.*0***	***20*.*1***	***162*.*4***	***184*.*4***	***35*.*3***	***75*.*6***	***9*.*7***	***7*.*6***

negative values occur where a commodity is imported then exported after processing.

**Table 2:** Comparison of the food sources supplying micronutrients available for consumption in the UK from domestic production and imports (1962 and 2010).

		Vitamin A (µg/capita/d)		Vitamin C (mg/capita/d)		Vitamin B12 (µg/capita/d)		Folate (µg/capita/d)		Zinc (mg/capita/d)		Iron (mg/capita/d)		Calcium (mg/capita/d)	
		Domestic	Import	Domestic	Import	Domestic	Import	Domestic	Import	Domestic	Import	Domestic	Import	Domestic	Import
Cereals	1962	16.1	23.8	2.1	3.1	0.2	0.3	46.4	58.8	1.2	1.5	3.0	3.7	143.7	154.0
	*2010*	*32*.*2*	*6*.*1*	*4*.*2*	*0*.*8*	*0*.*5*	*0*.*1*	*90*.*0*	*19*.*4*	*2*.*1*	*0*.*7*	*5*.*8*	*1*.*3*	*290*.*5*	*47*.*3*
Starchy roots	1962	0.0	0.0	18.8	1.8	0.0	0.0	34.1	3.2	0.4	0.0	0.4	0.0	11.7	1.1
	*2010*	*0*.*0*	*0*.*0*	*16*.*2*	*5*.*1*	*0*.*0*	*0*.*0*	*29*.*4*	*9*.*2*	*0*.*4*	*0*.*1*	*0*.*3*	*0*.*1*	*10*.*1*	*3*.*2*
Sugars	1962	0.5	4.1	0.0	0.0	0.0	0.0	0.0	0.0	0.0	0.1	0.1	0.7	2.6	21.7
	*2010*	*1*.*1*	*2*.*4*	*0*.*0*	*0*.*0*	*0*.*0*	*0*.*0*	*0*.*0*	*0*.*0*	*0*.*0*	*0*.*1*	*0*.*2*	*0*.*4*	*6*.*4*	*13*.*3*
Pulses	1962	0.7	1.4	0.0	0.0	0.0	0.0	0.9	3.0	0.1	0.2	0.1	0.3	1.3	4.5
	*2010*	*1*.*6*	*0*.*5*	*0*.*0*	*0*.*0*	*0*.*0*	*0*.*0*	*1*.*1*	*1*.*2*	*0*.*2*	*0*.*1*	*0*.*2*	*0*.*1*	*2*.*5*	*1*.*7*
Nuts, seeds	1962	0.0	0.0	0.0	0.1	0.0	0.0	-0.2	2.8	0.0	0.1	0.0	0.2	-0.4	4.4
	*2010*	*0*.*0*	*0*.*4*	*0*.*0*	*0*.*0*	*0*.*0*	*0*.*0*	*-0*.*7*	*6*.*3*	*0*.*0*	*0*.*3*	*0*.*0*	*0*.*4*	*0*.*7*	*11*.*7*
Vegetable oil	1962	1.0	1.3	0.0	0.0	0.0	0.0	0.0	0.0	0.0	0.0	0.0	0.0	0.0	0.0
	*2010*	*0*.*0*	*0*.*7*	*0*.*0*	*0*.*0*	*0*.*0*	*0*.*0*	*0*.*0*	*0*.*0*	*0*.*0*	*0*.*0*	*0*.*0*	*0*.*0*	*0*.*0*	*0*.*0*
Vegetables	1962	274.3	45.5	16.0	4.7	0.0	0.0	36.3	8.4	0.2	0.1	0.6	0.2	31.6	8.3
	*2010*	*176*.*3*	*233*.*0*	*10*.*6*	*18*.*1*	*0*.*0*	*0*.*0*	*24*.*4*	*35*.*2*	*0*.*2*	*0*.*2*	*0*.*4*	*0*.*7*	*23*.*1*	*30*.*9*
Fruit (incl juice)	1962	2.4	10.1	7.8	29.5	0.0	0.0	0.6	9.7	0.0	0.1	0.1	0.3	2.1	13.6
	*2010*	*0*.*2*	*23*.*8*	*0*.*7*	*78*.*0*	*0*.*0*	*0*.*0*	*-0*.*9*	*28*.*7*	*0*.*0*	*0*.*2*	*0*.*0*	*0*.*6*	*-0*.*3*	*32*.*6*
Meat (incl offal)	1962	349.8	190.3	1.4	1.0	1.8	1.3	26.5	13.9	2.0	1.3	1.3	0.8	12.4	8.9
	*2010*	*130*.*4*	*136*.*5*	*0*.*8*	*1*.*2*	*1*.*1*	*1*.*2*	*19*.*5*	*14*.*0*	*1*.*6*	*1*.*4*	*0*.*9*	*0*.*8*	*9*.*6*	*10*.*9*
Animal fat	1962	25.7	181.7	0.0	0.0	0.0	0.0	0.1	0.0	0.0	0.0	0.0	0.0	2.2	3.2
	*2010*	*29*.*9*	*45*.*7*	*0*.*0*	*0*.*0*	*0*.*0*	*0*.*0*	*-0*.*1*	*0*.*1*	*0*.*0*	*0*.*0*	*0*.*0*	*0*.*0*	*-0*.*7*	*3*.*0*
Eggs	1962	37.5	2.2	0.0	0.0	0.8	0.0	14.0	0.8	0.3	0.0	0.5	0.0	13.7	0.8
	*2010*	*24*.*7*	*3*.*8*	*0*.*0*	*0*.*0*	*0*.*5*	*0*.*1*	*9*.*2*	*1*.*4*	*0*.*2*	*0*.*0*	*0*.*3*	*0*.*1*	*9*.*0*	*1*.*4*
Milk & products	1962	177.5	41.8	7.4	1.7	3.6	0.8	45.0	10.6	2.4	0.6	0.3	0.1	657.3	154.9
	*2010*	*152*.*8*	*73*.*5*	*6*.*3*	*3*.*1*	*3*.*1*	*1*.*5*	*38*.*7*	*18*.*6*	*2*.*1*	*1*.*0*	*0*.*2*	*0*.*1*	*565*.*7*	*272*.*2*
Fish	1962	4.2	3.1	0.0	0.0	0.6	0.3	1.0	0.5	0.2	0.1	0.3	0.1	18.6	7.9
	*2010*	*1*.*0*	*7*.*7*	*0*.*0*	*0*.*0*	*0*.*1*	*1*.*0*	*0*.*1*	*2*.*3*	*0*.*0*	*0*.*3*	*0*.*0*	*0*.*5*	*-2*.*2*	*30*.*6*
Other foods	1962	-0.8	2.5	0.0	0.1	0.0	0.1	18.2	2.3	0.0	0.1	0.0	0.4	10.3	13.4
	*2010*	*-3*.*0*	*17*.*6*	*-0*.*1*	*1*.*5*	*0*.*0*	*0*.*1*	*13*.*3*	*7*.*6*	*-0*.*1*	*0*.*5*	*-0*.*3*	*1*.*9*	*-0*.*6*	*55*.*2*
**TOTAL**	**1962**	***888*.*9***	***508*.*1***	***53*.*6***	***42*.*0***	***7*.*0***	***2*.*8***	***223*.*0***	***114*.*1***	***6*.*9***	***4*.*3***	***6*.*7***	***6*.*9***	***907*.*1***	***370*.*0***
	***2010***	***551*.*7***	***551*.*7***	***38*.*8***	***107*.*9***	***5*.*3***	***4*.*0***	***223*.*9***	***144*.*1***	***6*.*7***	***4*.*9***	***8*.*2***	***7*.*1***	***913*.*8***	***514*.*0***

negative values occur where a commodity is imported then exported after processing.

Cereals were an important contributor to the supply of fibre, folate, calcium and iron, due to micronutrient fortification of cereal products such as flour and breakfast cereals and the high consumption of these foods. The drop in supply of domestic and imported vitamin A from meat products is almost solely due to the reduction in consumption of offal, especially liver, which is very high in vitamin A.

### Domestically produced and imported energy and macronutrients supply

**Fig 1:**
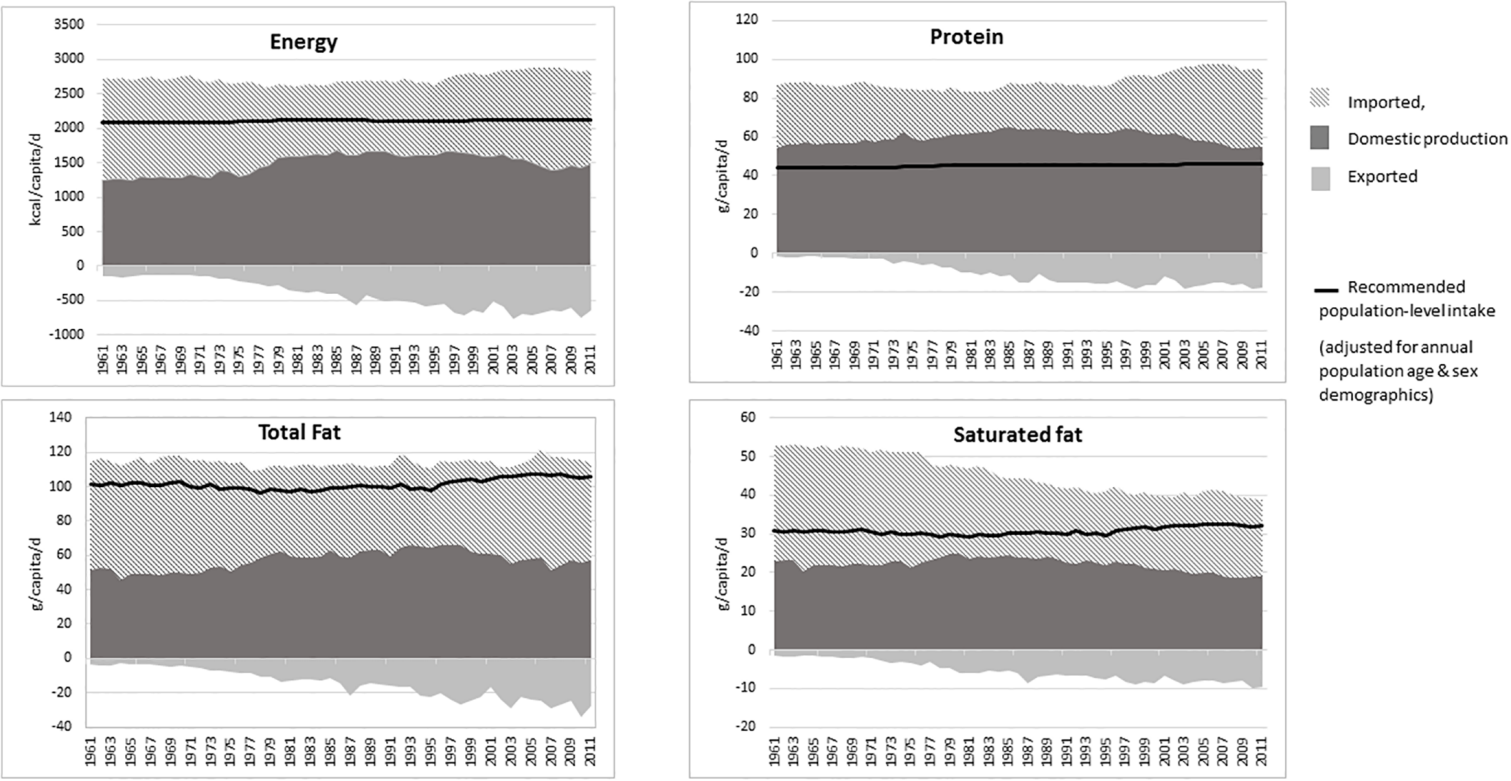
Supply of energy, protein and fats from domestic production, imported food and exported food.

Fig 1 shows the trend in domestic, imported and exported supply of energy, protein and fat in the food chain used for human consumption and compared to dietary requirements.

*Energy and protein*: Since the 1960s the total supply of energy in the UK has been greater than the population requirement, but the UK is reliant on imports to meet this requirement. The total energy supply has steadily increased, with energy supplies in 2010 about 210kcal/capita/d higher than at the start of the 1980s. If this increase in energy created a small persistent positive energy balance, it could have contributed to the increase in the prevalence of overweight and obesity in the UK. However, the increasing discrepancy between the food supply and reported energy intakes (consumed), which appear to have fallen slightly between the 1980s and 2010, is most likely to be due to an increase in household level food wastage over this time [31]. The supply of protein for human consumption has increased, particularly since the mid-1990s, and has been substantially higher than dietary requirements since 1961, even without imported foods. In 2010, approximately a third of protein came from plant-based products and even after correcting for the lower digestibility of plant-based protein [29], the total supply was still almost double the amount needed to satisfy the population requirements. Replacing the equivalent amount of energy from meat with non-protein foods, and adjusting for the lower digestibility of plant-based proteins, the supply of protein still meets the dietary requirements for the UK population.

*Fats*: Since the 1960s, the supply of total fat and saturated fat was higher than the current recommended population average intakes of 33% and 10% of total energy, respectively. The UK supply of total fat reduced very slightly from 38.0% to 36.6% of total energy between 1962 and 2010. While the reduction in saturated fat was greater, from 17.5% to 12.4% of total energy, this still exceeded the current population recommendation of 10% of total energy. The domestic supply of saturated fat has not changed over this time, but imports have reduced; this is largely due to the decrease in the import of animal fats.

**Fig 2:**
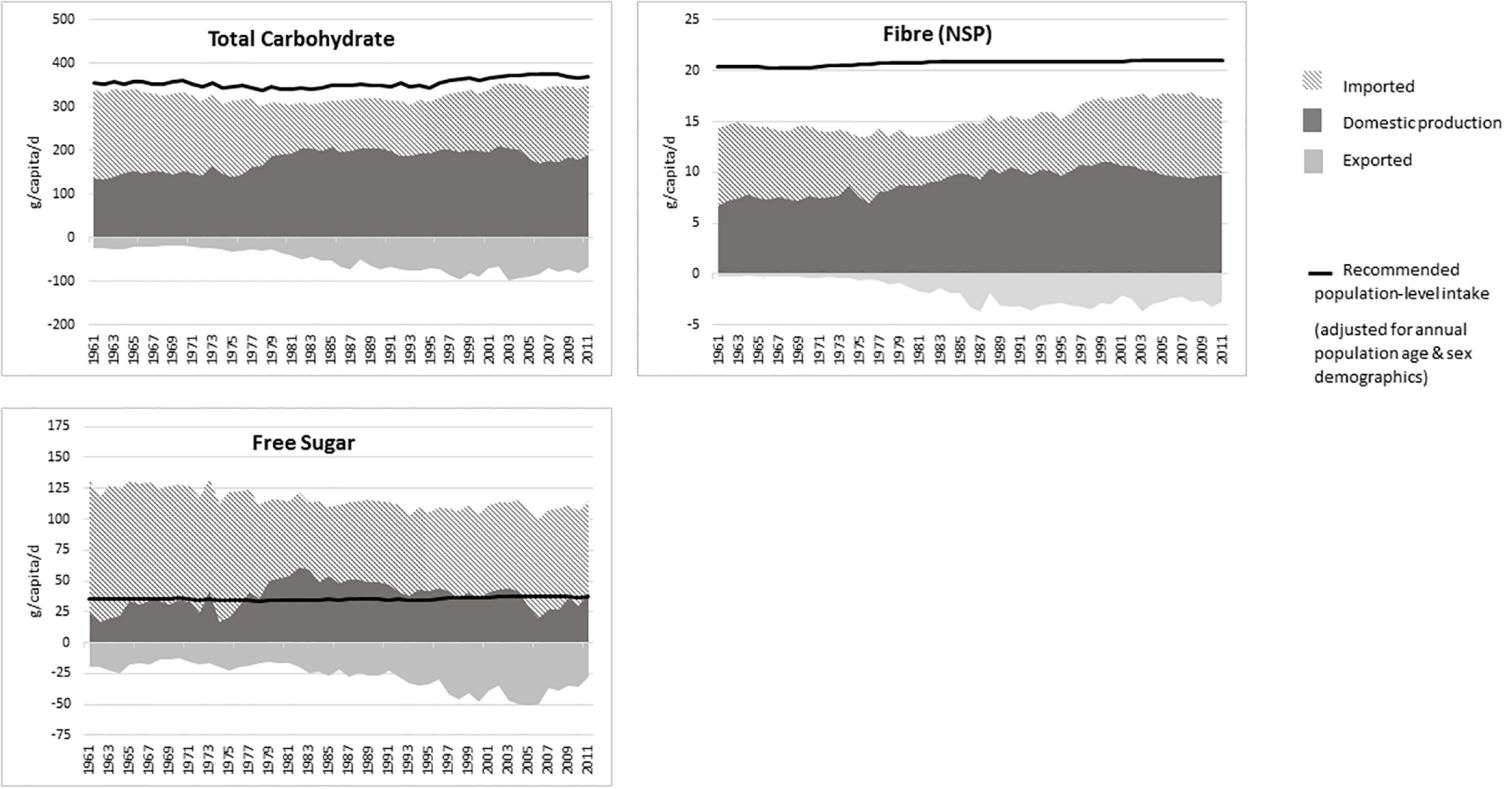
Supply of total carbohydrate, fibre and free sugar from domestic production, imported food and exported food.

Fig 2 shows the trend in domestic, imported and exported supply of total carbohydrate, fibre and free sugar in the food chain used for human consumption and compared to dietary requirements.

*Carbohydrates*: The supply of total carbohydrate has changed very little over the decades although a greater proportion now comes from domestically produced food. In 2010 the supply was estimated to be 347g/capita/d, which is 46% of total energy and below the recommended population average of 50% of total energy. The supply of free sugars accounted for about a third of total carbohydrates. Free sugars decreased from about 125g/capita/d (17.3% of total energy) in 1962 to 111g/capita/d (14.7% of total energy) in 2010, but this amount was still considerably above the recently revised recommended intake of 5% of total energy, which was reached by domestic supply alone.

The UK population dietary recommendation for fibre was recently increased to the equivalent of 23g/capita/d of NSP (30). As shown in Fig 2, although the total supply of dietary fibre for human consumption has increased over the decades it still falls short of population-weighted recommendation of 21g/capita/d. It is important to note that supply data were based on food purchased in the UK (e.g. bread, breakfast cereals) not raw commodities (e.g. wheat) and the results reflect the consumption of highly refined cereals in the UK. If all the cereal products consumed in the UK were unrefined (e.g. wholemeal flour, wholemeal bread, wholegrain breakfast cereals, whole-wheat pasta, brown rice) then the supply of NSP in 2010 would increase to around 23g/capita/d.

### Domestically produced and imported micronutrient supply

**Fig 3:**
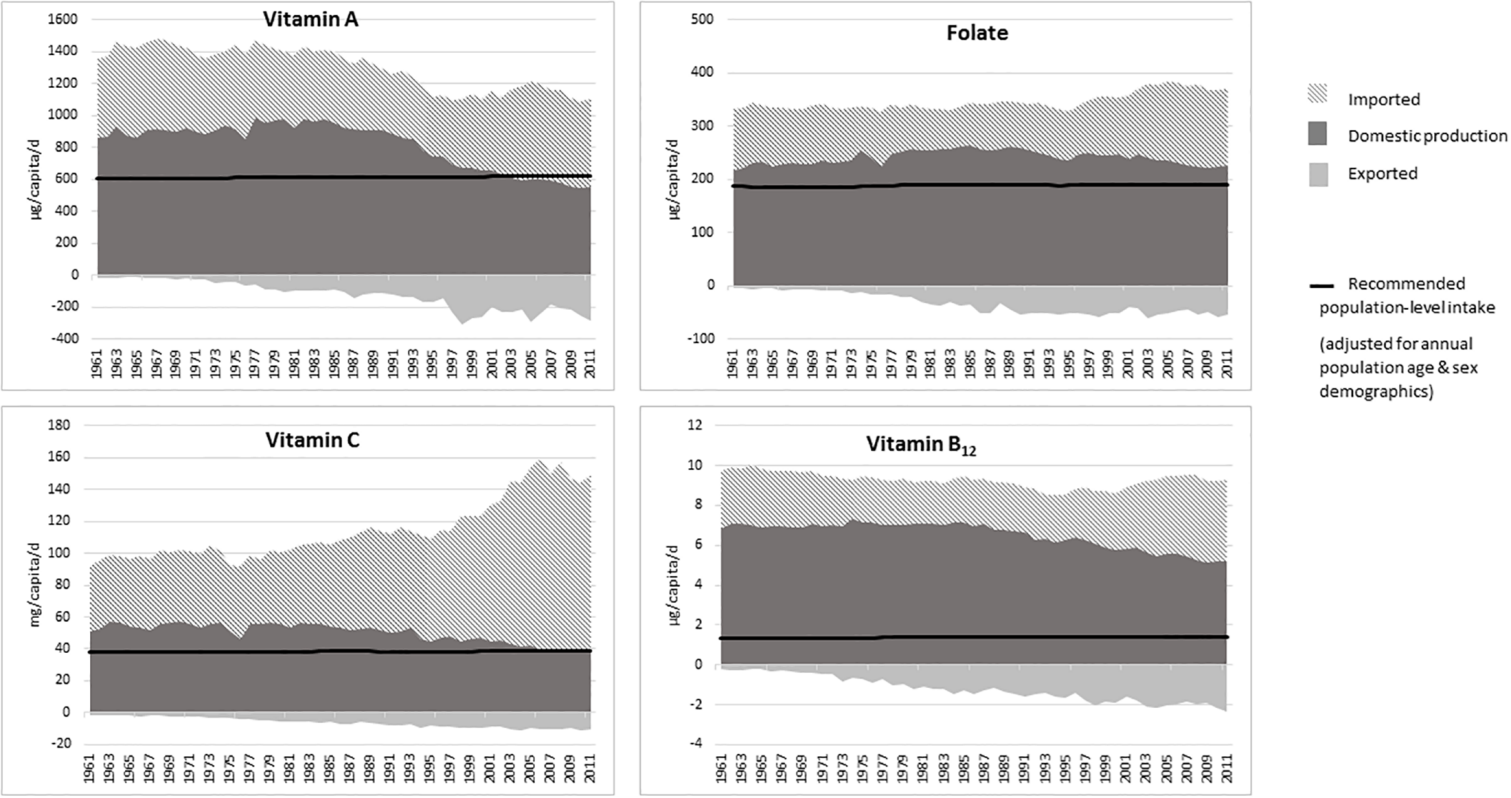
Supply of vitamins A, C and B_12_ and folate from domestic production, imported food and exported food.

**Fig 4:**
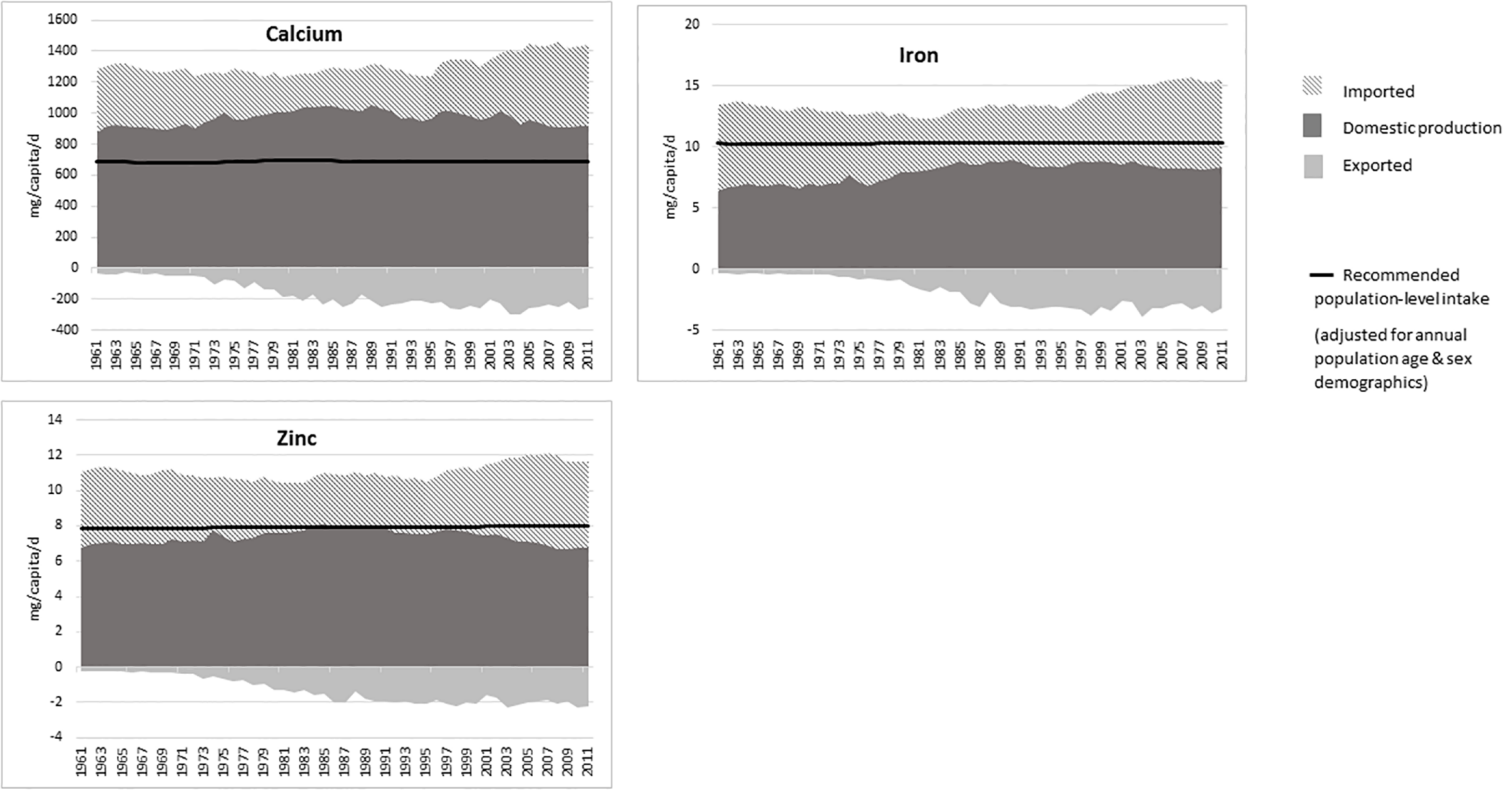
Supply of calcium, iron and zinc from domestic production, imported food and exported food.

The supply of micronutrients from domestically produced food and imported food along with exported food between 1961 and 2011 is shown in Figs 3 and 4. The total supply of these micronutrients was sufficient to meet the UK population requirements, with folate, vitamin B_12_ and calcium requirements being meet by domestically produced food alone, without the need for imports. The main source of vitamin B_12_ and calcium was domestic production of milk and milk products, while for folate it was from domestic production of cereal products, which are often fortified with minerals and vitamins. An adequate supply of iron and zinc was dependent on imports, and the UK has become increasingly dependent on imports to meet population requirements for vitamin A and vitamin C. The increase in domestic supply of iron followed the same trend as the supply of cereals in the UK. The main source of zinc was animal products (meat and milk) and more recently from cereals, due to fortification of cereal products. Cereals were a major source of many of the micronutrients, but it is noted that micronutrients in plant-based foods tend to be less bioavailable than in animal-based products [32].

In 2010, the dependency on imports to meet requirements of vitamins C and A was marginal. The greatest source of vitamin C was imported fruit, and of vitamin A was imported vegetables. Imports of both fruit and vegetables has increased while domestic production has decreased. Exports of all nutrients have increased over the decades.

Table 3 summarises the estimated percentage self-sufficiency in energy and nutrients to meet requirements in 2010, based on domestic production compared to the total supply of nutrients available in the UK.

**Table 3:** Percentage self-sufficiency for energy and nutrients based on the supply from domestic production (minus exports) and the total supply of food in the UK in 2010 compared to population-level requirements.

	Energy	Protein	Carbohydrate	Fibre	Calcium	Iron	Zinc	Vit A	Vit C	Vit B_12_	Folate
**Supply from domestic production**	69%	119%	49%	46%	133%	80%	84%	88%	101%	376%	119%
**Total supply**	133%	206%	92%	82%	207%	149%	146%	178%	381%	658%	196%

In summary, the results show that the UK was self-sufficient in the supply of protein, vitamin B_12_, calcium and folate from domestic production to satisfy population dietary requirements. While the UK has become marginally more self-sufficient in nutrients, it was still dependent on imports for 30% of energy, 20% of iron, 16% of zinc and 12% of vitamin A.

## Discussion

This paper presents a method for estimating national nutrition security and the dependency on imports to meet population nutritional requirements. Since the 1960s the supply of nutrients has been adequate to meet UK population-weighted dietary requirements, with the exception of fibre and total carbohydrates, but was dependent on imports for the supply of energy, iron and zinc to meet dietary needs. Over time, imports have become increasingly important for an adequate supply of vitamins A and C. The supply of free sugars, total fat and saturated fat exceeded current population recommended intakes, contributing to poor dietary intakes. The findings were consistent with nutrient patterns from the current UK National Diet and Nutrition Survey (NDNS) based on reported intakes, in which overconsumption of free sugars and saturated fat, and low intakes of fibre were reported [33]. The NDNS estimated that less than 5% of the UK population report dietary intakes that meet the minimum recommended intake of fibre.

This study showed that the supply of fibre was inadequate to meet population needs, but this does not necessarily mean that there was insufficient fibre in the food system, rather that there was a demand for highly processed foods, from which much of the fibre is removed. Wholegrain cereals are a good source of fibre, but in the UK 88% of the wheat used for bread flour is milled to white flour [34] and the fibre content of white flour is approximately a third of that of wholemeal flour [21]. Recalculating the estimated supply of fibre in the UK for human consumption assuming that all cereals were eaten as wholegrain showed that there would be sufficient fibre to meet population recommendations.

An important component in estimating nutrition security is including the impact of processing of food, as this can alter the nutrient composition of foods, with either positive or negative health benefits [35]. For example, processing whole fruit into fruit juice increases the free sugar content and in some cases reduces the fibre content, or the conversion of maize into corn syrup that results in removal of fibre and an increase in free sugars. In contrast, for some commodities micronutrients are lost in processing but then replaced by fortification of the product. In the UK, fortification of white flour with iron, calcium carbonate, niacin and thiamine has been mandatory since 1956, to compensate for losses during milling [36]. Micronutrients are also often added to foods, such as breakfast cereals and fat spreads, on a voluntary basis. As this study showed, a substantial proportion of the supply of the micronutrients come from fortified cereal products.

The effectiveness of fortification, however, depends on the bioavailability and absorption of the micronutrients [32,37]. Cereals and pulses are high in phytates, which reduce the bioavailability of zinc, iron and calcium, and the highest concentration of phytates tend to be in the bran where these micronutrients are naturally found. Similarly, haem iron, which comes from animal sources, is more bioavailable than non-haem iron found in plant-based food, though absorption can be enhanced by the foods it is eaten with (e.g. foods containing vitamin C).

To reduce the risk of climate change many countries, including the UK, need to shift from diets high in animal products to more plant-based diets, which could have implications in terms of the bioavailability of some nutrients [38]. Furthermore, it is predicted that climate change may not only alter productivity but also the nutrient composition of crops, which will have consequences for future nutrition security. Studies have shown that elevated levels of atmospheric CO_2_ are associated with lower concentrations of zinc and iron in crops such as wheat, rice and soybeans [39]. The assessment of future nutrition security will therefore need data on the nutrient composition of foods to be kept up to date.

In the UK, the supply of protein was estimated to be about double the population-level requirement, which is consistent with reported dietary intakes [33]. Meat consumption in the UK is high and strategies to mitigate climate change and reduce the risk of some diet-related diseases include reducing meat consumption [40,41]. This has driven both scientific and public debates around finding protein alternatives to replace meat [42,43], but in the UK where there is an adequate supply from non-meat sources suggests that there may not be a need to find high protein alternatives to meat. The supply of protein would still meet the dietary requirements for the UK population even if all meat were removed from the diet. Reducing meat consumption may have greater consequences for some of the micronutrients rather than for protein and perhaps this should be the focus rather than protein. Reducing meat consumption will also have implication further up the food chain with less animal feed needed, which could mean freeing up land for production of other commodities. It is sometimes assumed that the crops grown to feed to animals could be used for human consumption, but this may be over simplistic. From a nutrition security perspective it would be important to explore alternative crops that could be grown on the land to provide a more nutritionally adequate food supply, while recognising the limitations of the type of land and climate, as well as the cultural acceptability of alternative foods. This is an example of the type of scenarios where an interdisciplinary approach is needed to achieve environmentally sustainable nutrition security.

The risk of global shocks, such as extreme weather events, civil unrest, economic crisis, disruption of trade agreements and disease, as well as long-term impacts of climate change, drives debates about self-sufficiency and dependency on imports to meet population needs. In the UK, for example, the amount of fruit and vegetables imported has increased between 1962 and 2010 from 53% to 84%, with the supply mainly coming from the European Union (EU) [44]. These foods provide a significant supply of key nutrients, such as folate, fibre, vitamin C and vitamin A, to the UK. Trade and supply of sufficient fruit and vegetables is particularly relevant today for the UK. Trade with the EU currently occurs at relatively low transaction costs, but this could increase after the UK withdrawal from the EU and, depending on the nature of new trade agreements, could increase the costs to consumers [45]. This comes at a time when only 27% of the UK adult population report eating the recommended five portions a day [33]. On the other hand, UK domestic production of fruit and vegetables is low and while there might be potential to increase horticulture, whether this is economically sustainable or agriculturally feasible will need to be determined. Furthermore, the range of products available to people is likely to reduce if the supply of fruit and vegetables is limited to domestic production alone. Whether this reduced range of products will be acceptable to people is uncertain [46].

The methodology developed for estimating national nutrition security has some limitations. The FBS are estimates of the availability of food at a national level and may overestimate the nutrients actually consumed due to food wastage at the household level. Unlike previous studies [20,47], we made an attempt to adjust for household food waste. The accuracy of the nutrient dataset produced is dependent on matching foods in the FBS to those in the food composition tables, using limited descriptors. However, comparing the calculated values derived from UK composition dataset with those reported in the FBS for energy, protein and fat gave similar values and good agreement across the majority of commodities. Finally, nutrition security is dependent on more than just supply, as availability, access, utilisation and stability are equally important but the FBS do not provide information about the distribution of, and access to, nutrients within a country, at household or at an individual level. However, the methodology developed in this study could be used in future studies to assess national nutrition security and to estimate self-sufficiency in terms of a country’s dependency on imports to supply their population requirements. This goes beyond previously published studies [10,14], which tend to only consider self-sufficiency in terms of energy.

## Conclusions

This study developed a method to assess national nutrition security rather than food security, including nutrients that are abundant and those that are deficient at a national level, and the dependency on imports to meet population requirements. The results show findings for the UK but the same methodology could be applied to any country with detailed food composition data. The study also provides an alternative view of self-sufficiency by looking at the dependence on imports to meet population-level nutritional requirements, rather than viewing self-sufficiency from an economic perspective. The concept of self-sufficiency could be broadened from a unidimensional perspective to include multiple factors, such as economics and nutrition security. As an approach to determine nutrition security at a national level, this study provides a method to monitor the progress toward delivering some of the sustainable development goals in terms of a national supply of sufficient nutrients to meet population requirements.

## Supporting information

S1 Fig Correlation of food commodity data comparing the FBS and M&W derived values for energy, protein and total fat. (TIF)

S1 Table The aggregate of food balance sheets food groups used in the analysis. (DOCX)

S1 Data The nutrient intake data for each food group in the food balance sheet. (XLSX)
