# Supplementary figures and images for: Assessing national nutrition security: The UK reliance on imports to meet population energy and nutrient recommendations
Source: PLoS One. 2018 Feb 28;13(2):e0192649. doi: 10.1371/journal.pone.0192649 (PMC5831084; doi:10.1371/journal.pone.0192649)

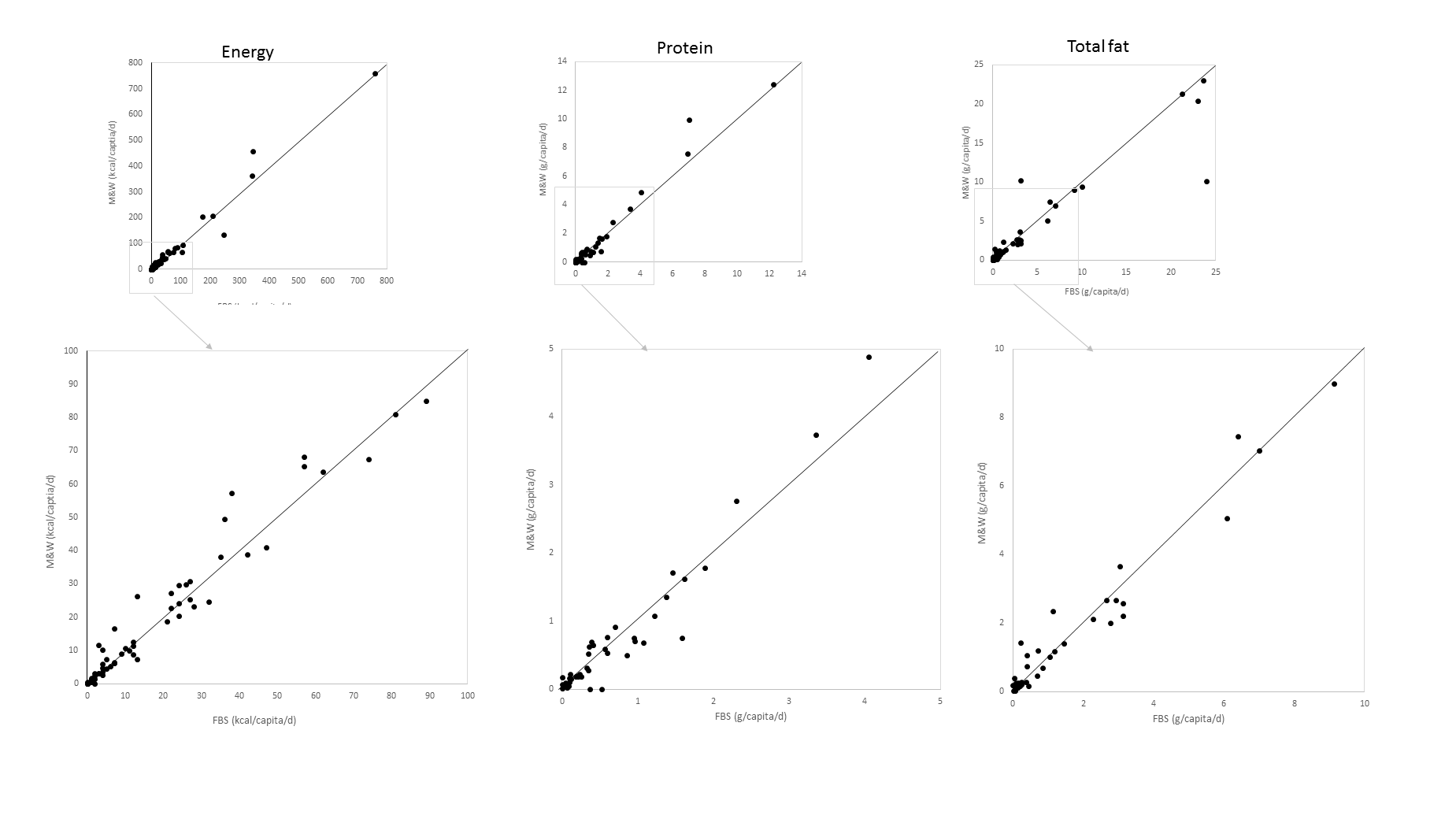

Supplement: S1 Fig — (TIF) [file pone.0192649.s001.tif]
