# Supplementary material for: Assessing national nutrition security: The UK reliance on imports to meet population energy and nutrient recommendations
Source: PLoS One. 2018 Feb 28;13(2):e0192649. doi: 10.1371/journal.pone.0192649 (PMC5831084; doi:10.1371/journal.pone.0192649)
Supplement: S1 Table — (DOCX) [file pone.0192649.s002.docx]

Table S1: The aggregate of food balance sheets food groups for the analysis

| **Food groups** | **Food balance sheets foods** |
| --- | --- |
| Cereals | Wheat and products, Rice (Milled Equivalent), Barley and products, Maize and products, Rye and products, Oats, Millet and products, Sorghum and products, Other cereals |
| Starchy roots | Potatoes and products, Sweet potatoes, Cassava and products, Roots (Other) |
| Sugars | Sugar (Raw Equivalent), Sweeteners, Honey, Sugar cane, Sugar beet |
| Pulses | Beans, Peas, Other pulses and products |
| Nuts, seeds | Nuts and products, Groundnuts (Shelled Eq), Sunflower seed, Sesame seed |
| Vegetable oil | Soyabean Oil, Groundnut Oil, Sunflowerseed Oil, Rape and Mustard Oil, Palm kernel Oil, Palm Oil, Coconut Oil, Sesameseed Oil, Olive Oil, Maize Germ Oil, Oilcrops Oil, Other |
| Vegetables | Tomatoes and products, Onions, Vegetables (Other) |
| Fruit (incl. juice) | Oranges, Mandarines, Lemons, Limes and products, Grapefruit and products, Citrus (Other), Bananas, Plantains, Apples and products, Pineapples and products, Dates, Grapes and products (excl wine), Fruits (Other) |
| Meat (incl offal) | Bovine Meat, Mutton & Goat Meat, Pigmeat, Poultry Meat, Meat ( Other), Offals |
| Animal fat | Butter, Ghee, Cream, Other animals fats, Fish, Body Oil, Fish, Liver Oil |
| Eggs | Eggs |
| Milk & products | Milk and milk products (Excluding Butter); milk, cheese, yoghurt |
| Fish | Freshwater Fish, Demersal Fish, Pelagic Fish, Marine Fish (Other), Crustaceans, Cephalopods, Molluscs (Other), Aquatic Animals (Others) |
| Other foods | Coffee and products, Cocoa Beans and products, Tea (including mate), Pepper, Pimento, Cloves, Spices (Other), Wine, Beer, Beverages (Fermented), Beverages (Alcoholic), Infant food, Miscellaneous |
